# Practical strategies to manage cancer patients during the COVID-19 pandemic: Saudi Oncology Pharmacy Assembly Experts recommendations

**DOI:** 10.1177/1078155220935564

**Published:** 2020-06-24

**Authors:** Majed Alshamrani, Atika AlHarbi, Nora Alkhudair, Fouad AlNajjar, Mansoor Khan, Abdulaziz Ben Obaid, Amr Khardaly, Eshtyag Bajnaid, Hadeel Samarkandi, Aeshah AlAzmi, Salman Alabdali, Mohammed AlNahedh

**Affiliations:** 1 Saudi Oncology Pharmacy Assembly, Riyadh, Saudi Arabia; 2 Pharmaceutical Care Services, Ministry of National Guard Health Affairs, Jeddah, Saudi Arabia; 3 Department of Clinical Pharmacy, College of Pharmacy, King Saud University, Riyadh, Saudi Arabia; 4 Pharmaceutical Care Services Department, Comprehensive Cancer Center, King Fahad Medical City, Riyadh, Saudi Arabia; 5 Drug Policy and Control Department, Assistant Deputyship for Therapeutic Supportive Services, Ministry of Health, Riyadh, Saudi Arabia; 6 Clinical Pharmacy Department, King Abdullah Medical City, Mecca, Saudi Arabia; 7 Department of Pharmaceutical Care, Oncology Pharmacy Services, King Faisal Specialist Hospital & Research Center, Riyadh, Saudi Arabia; 8 King Fahad Hospital, Ministry of Health, Almadina, Saudi Arabia

**Keywords:** Coronavirus, COVID-19, cancer, pandemic, SOPA

## Abstract

**Purpose:**

During COVID-19 pandemic, cancer patients are considered one of the most vulnerable to infection since they tend to have advanced age, multiple comorbidities, and are often immunosuppressed by their cancer or therapy. Hence, the Saudi Oncology Pharmacy Assembly has issued recommendations to reduce the frequency of cancer patients’ visits to oncology centers during the pandemic while maintaining the access to cancer therapy and minimize the risk of exposure to coronavirus disease.

**Materials and methods:**

A qualitative methodological approach was conducted in April 2020 using a virtual panel discussion for collection of recommendations.

**Results:**

A total of 12 expert oncology pharmacy practitioners shared their knowledge and experiences in managing oncology patients during the COVID-19 pandemic. The participants recognized many fundamental recommendations that were already applied in many cancer centers since the start of the COVID-19 outbreak. On that basis, the panelists developed eight practice-related recommendations for action, with a main focus on cancer treatment modification.

**Conclusions:**

In conclusion, delivering cancer care during the COVID-19 pandemic carries significant challenges. This paper addressed suggestions to properly manage cancer patients during difficult times. Implementing changes in practice mandates a national collaborative effort from different sectors to guarantee the quality and continuity of care. The SOPA expert panel developed these recommendations, to ultimately contribute in maintaining access to cancer therapy while minimizing the risk of COVID-19 exposure.

## Introduction

A novel coronavirus was isolated and reported in Wuhan, China, in January 2020. It is now termed as severe acute respiratory syndrome coronavirus 2 (SARS-CoV-2), causing a respiratory disease called coronavirus disease 2019 (COVID-19) in infected individuals. Early estimates of COVID-19 case fatality rates were about 2%, increasing up to 15% in patients aged 80 years or over. COVID-19 disease has spread rapidly worldwide, meeting conventional definitions of a pandemic.^1^

In Saudi Arabia, the first case of the COVID-19 pandemic was confirmed on 2 March 2 2020 from a traveler. A widespread transmission is still ongoing, and precautionary actions took place to control the disease such as suspension of many government agencies, suspension of domestic and international flights, closure of schools and universities.

The outbreak of COVID-19 has become a public health crisis of major global concern. Given the immunosuppressive state caused by cancer itself and anticancer treatments, patients with cancer may be at a higher risk of COVID-19 infection.^2^ Moreover, there are additional risk factors for severe COVID-19 disease in cancer patients, such as malignancy in infants and children younger than five years, advanced age, poor performance status, organ dysfunction, and comorbidities.^3–5^ Early COVID-19 outcome statistics suggested a case fatality rate of 5.6% among patients with cancer.^1^ A study reported that patients with cancer had a higher risk of severe events with COVID-19 infection, such as admission to the intensive care unit, invasive ventilation, or death.^2^,^6^ Therefore, a multidisciplinary approach and open communication between health care providers and patients should be maximized. Oncology physicians need to reevaluate the risks versus benefits of cancer treatments, balancing the risks of disease progression against the risk of COVID-19 infection can be considered on a case-by-case basis, while ensuring that all actions taken are fair and transparent.^7^

In response to COVID-19 pandemic, the National Comprehensive Cancer Network (NCCN) and the European Society for Medical Oncology (ESMO) experts published their insights and advice on how to continue providing the best cancer care during the COVID-19 pandemic. They stressed on the significance of keeping open communication between administrators, staff, patients, caregivers, and the general public. They recommended creating an incident command structure (ICS) to provide early coordination of institution-wide efforts and to respond to changing information quickly. They highlighted the need to remain flexible and ready for unexpected challenges.^8^,^9^

The purpose of this paper is to discuss some practical recommendations to reduce the frequency of cancer patients’ visits to oncology centers while maintaining access to cancer therapy and minimizing the risk of exposure to COVID-19 endorsed by Saudi Oncology Pharmacy Assembly (SOPA) Experts (Table 1).

**Table 1.** Executive summary of SOPA practical strategies to manage cancer patients during the COVID-19 pandemic.

Recommendation	Examples
1. Delaying adjuvant chemotherapy within the recommended range of treatment initiation	• Delaying adjuvant chemotherapy for a maximum of 12 weeks in early stages breast cancer (excluding TNBC & HER2-positive breast cancer) • Delaying adjuvant chemotherapy for a maximum of 8 weeks in colorectal and gastric cancer
2. Use of extended dosing schedule of cancer therapy	• Extended dosing schedule of ICPIs • Use of every 3 weeks schedule of taxanes in patients with advanced breast cancer • Use of every 12 weeks BMAs for bone metastases • Use of every 12 and 24 weeks GnRHa for advance breast and prostate cancer, respectively • Use of every 12 weeks VCR/DEX pulses for pediatric with SR B-ALL according to NCI without CNS or testicular leukemia, unfavorable genetic characteristics • Use of pegaspargase for pediatric and adult ALL without previous history of *E. coli* L-asparaginase allergy
3. Switching from intravenous chemotherapy to oral or subcutaneous route of administration	• Shifting patients to from IV to PO chemotherapy (e.g. etoposide, cyclophosphamide, topotecan and vinorelbine) • Replacing IV 5-FU with PO capecitabine • Use oral chemotherapy for maintenance therapy in multiple myeloma; lenalidomide in standard-risk patients and ixazomib in high-risk patients (If PO ixazomib is not available and bortezomib SQ cannot be provided, lenalidomide PO can be considered, with close monitoring). • Using the SQ route for rituximab, trastuzumab, and daratumumab.
4. Home administration of chemotherapy and supportive care therapy	• Ideal chemotherapy medications that can be administered at home can include the following: ○ Medications administered SQ: azacitidine, bortezomib, cladribine, cytarabine (palliative setting), SQ rituximab, SQ trastuzumab, SQ daratumumab ○ Medications administered intravenously through IV push or short infusions (e.g. vinca alkaloids) ○ Medications administered intravenously through IV pump (e.g. 5-FU and blinatumomab) • Supportive care medications can be given orally instead of intravenously as: ○ IV hydration can be given orally with proper patient education or intravenously at home through elastomeric infusion pumps when needed. ○ Oral antiemetics. ○ Growth factors and oral antibiotics can be used for patients receiving high-risk chemotherapy as primary prophylaxis for FN and empiric oral antibiotics in clinically stable patients with FN. ○ Oral sodium bicarbonate with or without acetazolamide if needed, and oral leucovorin with HDMTX with proper monitoring. ○ Oral mesna at home can be given with ifosfamide or high-dose cyclophosphamide administration. ○ In non-acute cases, electrolytes should be replaced orally when feasible.
5. Delay stem cell transplants if medically feasible	• If the patient disease risk allows, postpone all procedures related to HSCT (mobilization, collection, and conditioning) ○ Examples of autologous transplants: multiple myeloma and low-grade lymphomas. ○ Examples of allogeneic transplant: MRD negative ALL, intermediate-risk AML tolerating consolidation, MDS patients tolerant to transfusion and without excessive blasts, MF patients tolerating transfusions, and benign hematology indications (e.g. SCD, thalassemia) • If patient with high risk/aggressive disease, and the transplant cannot be postponed ○ Patient need to be tested for COVID-19 ○ Patient who doesn’t have respiratory symptoms and no exposure to COVID-19, proceed to transplant after self-isolating for 14 days. ○ Patient who has had close contact with a COVID-19 patient, BMT procedures should be deferred for 14–21 days from the date of last contact. The patient needs to have two negative PCR results one week apart before proceeding with any procedures. ○ Patients who are diagnosed with COVID-19, transplant procedures should be deferred until the patient becomes asymptomatic and has two negative PCR results at least one week apart, with a minimum of a 14-day deferral. ○ The use of lowest possible intensity regiment is used for such patient.
6. Consider intermittent chemotherapy or treatment discontinuation for eligible patients	• Chemotherapy holidays in metastatic diseases setting after multidisciplinary tumor board discussion and according to patient preference • Discontinuation of TKIs therapy in adult CML patients who achieve and maintain MMR with careful monitoring
7. Activating telemedicine for managing stable cancer patients on oral chemotherapy	Activating virtual oncology clinics for managing stable patients including the ones on oral chemotherapy (e.g. CML, CLL, lung cancer, breast cancer, renal cancer, prostate cancer, and HCC)
8. Applying innovative ideas to minimize patients visits to the pharmacy	• Sending medications by postal carriers to patients’ homes and use of a drive-through medications collection area • Providing more medications supply to chronic and stable cancer patients while maintaining pharmacy stock • Collaboration between pharmacy departments at oncology centers to continue providing treatments of patients who are affected by the travel restriction • Activation of 24 h hotline/on-call service run by oncology pharmacist for consultation and inquires related to cancer therapies.

TNBC: triple-negative breast cancer; ICPIs: immune checkpoint inhibitors; BMAs: bone modifying agents; LHRH: luteinizing hormone-releasing hormone; GnRHa: gonadotropin-releasing hormone agonist; SR B-ALL: standard-risk B cell acute lymphoblastic leukemia; NCI: National Cancer Institute; VCR/DEX: vincristine/dexamethasone; TKI: tyrosine kinase inhibitors; CML: chronic myeloid leukemia; MMR: major molecular response; CLL: chronic lymphocytic leukemia; HCC: hepatocellular cancer; SQ: subcutaneous; HDMTX: high-dose methotrexate; FN: febrile neutropenia; MRD: minimal residual disease; AML: acute myeloid leukemia; MDS: myelodysplastic syndromes; MF: myelofibrosis; SCD: sickle cell disease.

## Recommendation #1. Delaying adjuvant chemotherapy within the recommended range of treatment initiation

The benefits of adjuvant chemotherapy on disease progression and overall survival have been clearly demonstrated in many solid tumors. However, the optimal timing of chemotherapy initiation is still a matter of debate. For instance, in breast cancer, the initiation of adjuvant chemotherapy is typically started within four to eight weeks following surgery. In a large observational study, adverse outcomes are associated with delaying initiation of adjuvant chemotherapy 91 or more days in different breast cancer subtypes except for Triple-Negative Breast Cancer (TNBC), where the delay in chemotherapy was associated with detrimental effects.^10^ Moreover, ESMO considers HER2-positive breast cancer as a high priority for initiating chemotherapy as the delay in chemotherapy may be associated with detrimental effects.^11^ In major gastrointestinal malignancies, a systematic review and meta-analysis concluded that starting adjuvant chemotherapy within six to eight weeks post-surgery is associated with a significant survival benefit for colorectal and gastric cancer. The delay of adjuvant chemotherapy in colorectal cancer (>6–8 weeks post-surgery) was associated with a statistically significant increased risk of death (hazard ratio (HR) = 1.27, 95% confidence interval (CI) 1.21–1.33; *p* < 0.001). Similarly, for gastric cancer, delaying adjuvant chemotherapy more than six to eight weeks was associated with inferior overall survival (HR = 1.2, 95% CI 1.04–1.38; *p* = 0.01).^12^

In the light of the above supporting evidence of delaying adjuvant chemotherapy to mitigate the risk of COVID-19 exposure, delaying adjuvant chemotherapy for a maximum of 12 weeks in early stages breast cancer (excluding TNBC & HER2-positive Breast Cancer), and for not more than 8 weeks in colorectal and gastric cancer can be considered.

## Recommendation # 2. Use of extended dosing schedule of cancer therapy

### Extended dosing schedule of immune checkpoint inhibitors

Since their introduction, immune checkpoint inhibitors have dramatically changed the treatment landscape in oncology, offering durable responses and improved survival in many cancer types.^13^ Nivolumab and pembrolizumab have been widely prescribed for many patients across several tumor types. Nivolumab was approved at a dose of either 240 mg or 3 mg/kg every two weeks and pembrolizumab was approved at a dose of either 200 mg or 2 mg/kg every three weeks. An alternative extended dosing regimen based on pharmacokinetic studies for both medications was offered to provide convenience and flexibility to patients and prescribers. Nivolumab 480 mg every 4 weeks (Q4W) was approved in the European Union, the United States, and several other markets across numerous tumor types. Its approval was supported by quantitative efficacy/safety analyses bridging to 3 mg/kg every 2 weeks (Q2W).^14^ Additionally, pembrolizumab 400 mg every 6 weeks (Q6W) is currently approved by the European Union for monotherapy indications and for monotherapy and combination therapy indications by the U.S. Food and Drug Administration (FDA). The 400 mg Q6W dosing regimen had similar predicted exposures compared to those achieved at 200 mg Q3W.^15^

We recommend switching all patients on monotherapy nivolumab and pembrolizumab considering the significant number of cancer patients on ICPIs and the risk of exposure to COVID-19 in the infusion centers to 480 mg every four weeks and 400 mg every six weeks, respectively.

### Use of every three weeks schedule of taxanes in patients with advanced breast cancer

Taxanes have remained a cornerstone of breast cancer treatment over the past decades, improving the survival in both early and late-stage disease. All three formulations (docetaxel, paclitaxel, and nab-paclitaxel) are frequently administered either weekly or every three weeks (Q3W) schedule. Several studies evaluated different doses and schedules in different disease stages. A meta-analysis of 11 randomized controlled trials comparing Q3W versus weekly taxanes regimens in advanced breast cancer found that objective response rate (ORR) was better with Q3W schedule paclitaxel, whereas overall survival (OS) was longer in patients on weekly schedules. For docetaxel, no differences were found between schedules in terms of ORR, disease-free survival (DFS), and OS.^16^,^17^ Weekly paclitaxel is generally preferred in advanced breast cancer, but it is less convenient and has comparable efficacy to Q3W docetaxel.

Since the number of breast cancer patients visiting the infusion centers is very high, we recommend using docetaxel every three weeks rather than weekly paclitaxel in patients with advanced breast cancer to combat the risk of COVID-19.

### Use of every 12 weeks bone modifying agents for bone metastases

Using BMA in patients with bone metastases extended interval strategy (every 12 weeks versus every 3–4 weeks) of zoledronic acid should be encouraged across different types of malignancies.^18–20^ Additionally, switching patients to subcutaneous (SQ) denosumab, given every three months, is a convenient and effective option. However, it might have some financial burden depending on the clinical setting.^21^,^22^

### Use of every 12–24 weeks gonadotropin-releasing hormone analogues

Leuprolide, triptorelin and goserelin are classified as gonadotropin-releasing hormone analogues. They are commonly used in oncology clinical practice to treat patients with prostate or breast cancer. These three medications are available in sustained-release formulations with different concentrations. Their frequency of administration can be reduced to every three or six months’ injections. Therefore, it reduces the frequency of visits and prevents additional financial burdens.^23–26^ In prostate cancer, four approved doses and formulations of leuprolide are available and can be administered as intramuscular (IM) or subcutaneous (SQ) injections; leuprolide 7.5 mg monthly, 22.5 mg every 12 weeks, 30 mg every 16 weeks, and 45 mg every 24 weeks. Leuprolide 3.75 mg and goserelin 3.6 mg every four weeks are used to treat premenopausal patients with advanced or recurrent, hormone-positive breast cancer. An evidence of three leuprolide formulations (7.5 mg every 4 weeks, 11.5 mg every 12 weeks, and 22.5 mg every 24 weeks) concluded that the three formulations offered comparable efficacy and safety in prostate cancer. Furthermore, randomized studies compared subcutaneous injection of goserelin 10.8 mg every 12 weeks (Q12W) and 3.6 mg every 4 weeks (Q4W) in premenopausal patients with advanced or recurrent, hormone-positive breast cancer and in advance prostate cancer. The studies revealed that Q12W goserelin has similar efficacy and safety as that of Q4W administration.^24^,^25^ Triptorelin is another gonadotropin-releasing hormone analogues used to achieve medical castration levels of testosterone in patients with locally advanced or metastatic prostate cancer at a dose of 3.75 mg intramuscular (IM) every 4 weeks, 11.25 mg IM every 12 weeks or 22.5 every 24 weeks. One study has demonstrated that triptorelin every 12 or 24 weeks is efficacious and well-tolerated as every 4 weeks of administration.^26^

### Use of every 12 weeks vincristine/dexamethasone pulses for pediatric with standard-risk B cell acute lymphoblastic leukemia according to National Cancer Institute without CNS or testicular leukemia, unfavorable genetic characteristics

The use of vincristine/steroid monthly pulses, methotrexate (MTX), and mercaptopurine is the cornerstone in the maintenance phase of pediatric B-ALL treatment. A randomized controlled trial revealed that the disease-free survival (DFS) for the SR-BALL of patients receiving VCR/DEX pulse every 4 weeks versus every 12 weeks was 94.1% ±1.0% and 95.1% ±0.9%, respectively, while the 5-year OS for patients receiving VCR/DEX pulse every four weeks versus every 12 weeks was 98.3% ±0.5% and 98.6% ±0.5%, respectively.^27^

Since the number of pediatric SR-BALL patients visiting the infusion centers for VCR/DEX pulse is very high, we recommend using VCR/DEX pulse every 12 weeks rather than every 4 weeks to combat the risk of COVID-19 without affecting patient outcomes.

### Use of pegaspargase for pediatric and adult acute lymphoblastic leukemia without previous history of E. coli L-asparaginase allergy

Pegaspargase is part of antineoplastic combination therapy for treating ALL in children, adolescents, and young adults (AYA). It has a longer half-life (5.7 days) in comparison to *E. coli* L-asparaginase. It provides comparable efficacy to *E. coli* L-asparaginase with a decreased incidence of hypersensitivity reactions.^28^ Since one dose of pegaspargase is equivalent to six to nine doses of *E. coli* L-asparaginase, it is estimated to be a cost-effective option compared to *E. coli* L-asparaginase for the treatment of ALL in pediatric and adults patients. Therefore, we recommend using pegaspargase, if the patient had no previous history of *E. coli* L-asparaginase allergy, to combat the risk of COVID-19 without affecting patient outcomes.

## Recommendation # 3. Switching from intravenous chemotherapy to oral or subcutaneous route of administration

### Oral administration

Oral (PO) chemotherapy agents are preferred among oncology patients and practitioners due to their simple and convenient administration methods. However, they carry similar risks and adverse events of conventional therapies. Moreover, it requires a comprehensive patient education, frequent and proper dose adjustments, and close monitoring.^29–31^ In the light of the COVID-19 pandemic, the ESMO recently suggested switching stable patients from intravenous to oral route whenever possible.^9^ Limiting the patient’s exposure, reducing the number of patients in infusion centers and improving the utilization of healthcare resources can be achieved by switching to oral agents. Several conventional agents are available in oral dosage forms which have been proven to be equivalent to intravenous (IV) preparations through pharmacokinetic equivalence studies. The downside of using oral agents is the difficulty in ensuring patient adherence, especially in patients with polypharmacy and low health literacy.^32–34^

• Shifting patients to oral chemotherapy was studied extensively in numerous solid malignancies (e.g. vinorelbine, etoposide, cyclophosphamide, topotecan and capecitabine).
 ○ Oral vinorelbine (60 mg/m^2^ or 80 mg/m^2^) instead of IV (25 mg/m^2^ or 30 mg/m^2^) was used in patients with metastatic breast cancer (MBC) to the bone. Oral vinorelbine was well tolerated with 8.2 months median progression-free survival (PFS) and 35.2 months OS.^35–37^
 ○ Replacing IV etoposide with oral etoposide was studied in several hematological and solid malignancies. The reduced bioavailability (∼50%) of the oral formulation warrants doubling the dose oral etoposide when switching from IV to oral etoposide.^38–41^
 ○  Capecitabine is an oral fluoropyrimidine that can substitute IV 5-fluorouracil (5-FU).^42–44^ A meta-analysis included participants with curative and palliative intent concluded that there was no difference in ORR and OS between PO versus IV when treating colorectal cancer.^45^ Additionally, in MBC, oral capecitabine can be used alone or in combination or as a substitution for 5-fluorouracil with other PO/IV/SQ agents in MBC.^46^
• Maintenance therapy following autologous stem cell transplant in patients with multiple myeloma is currently the standard of care. However, using oral agents as lenalidomide in standard-risk patients and ixazomib in high-risk patients should be encouraged during the COVID-19 outbreak.^47–50^ Additionally, switching high-risk patients to lenalidomide PO can be considered, with close monitoring, if PO ixazomib is not available and bortezomib SQ cannot be provided.^51^

### Subcutaneous administration

Subcutaneous (SQ) administration of chemotherapy agents regained recent popularity with the approval of SQ monoclonal antibodies. The SQ formulations are easier in administration with a similar pharmacokinetic profile.^52^,^53^ However, there are some downsides of SQ administration including; local site reactions, delayed absorption, and limited volume.^54^

- Trastuzumab was approved to be administered subcutaneously in Europe since 2016. A fixed-dose of 600 mg every three weeks without loading dose was found to be non-inferior to conventional dosing in MBC.^55–57^ Using the SQ route is a convenient option for patients and providers. It has short administration and monitoring time, improve bed utilization, reduce nurse observation and administration time.^53^
- Rituximab was approved in 2017 by the US FDA, to be administered SQ at a flat dose of 1400 mg. The SQ formulation can be administered in 5 to 7 min in contrast to the IV formulation which usually takes up to 4 to 6 h to be completed. Thus, switching patients with the approved SQ indications after at least one dose of IV rituximab guarantees a similar efficacy and safety. Additionally, SQ formulation can improve patient’s satisfaction and avoid prolonged administration time during the pandemic.^58^
- The SQ daratumumab formulation granted the US FDA approval for multiple myeloma based on non-inferiority phase III trial, recently. The formulation considerably reduces treatment burden, as the fixed-dose of 1800 mg injection is administered in approximately 3 to 5 min, offering patients a more convenient treatment experience.^59^

## Recommendation # 4. Home administration of chemotherapy and supportive care therapy

Two studies were outlining different experiences of home administration of chemotherapy in both the adult and pediatric settings. The studies revealed an improvement in patient and/or patient’s caregiver quality of life. Furthermore, home administration of chemotherapy allows for better utilization of resources without affecting efficacy and safety.^60,61^ To limit patient visits to hospitals and clinics during the COVID-19 pandemic, and possibly beyond, providers should strongly consider administering chemotherapy at home when feasible. ASCO urges providers to “Consider whether home infusion of chemotherapy drugs is medically and logistically feasible for the patient, medical team, and caregivers”.^62^ This is more practical when considering supportive care medication (e.g. hydration, etc.).

When considering administering chemotherapy at home, this can be done by either qualified nurses or the patient’s caregiver after proper education and training, as demonstrated in the literature.

Ideal chemotherapy medications to be administered at home can include the following:

- Medications administered SQ:
   ○  Azacitidine
   ○  Bortezomib
   ○  Cladribine
   ○  Cytarabine (in the palliative setting)
   ○ Subcutaneous rituximab (subsequent doses for patients who do not have a prior history of infusion reactions)
   ○  Subcutaneous trastuzumab (subsequent doses for patients who do not have a prior history of infusion reactions)
   ○  Subcutaneous daratumumab
- Medications administered intravenously through IV push or short infusions (e.g. vinca alkaloids).
- Medications administered intravenously through IV pump (e.g. 5-FU and blinatumomab).

In addition to chemotherapy medications, several supportive care medications can be given at home or can be given orally instead of intravenously. Examples include:

- IV hydration can be given orally with proper patient education or intravenously at home through elastomeric infusion pumps when continuous hydration is needed.
- Oral antiemetics, rather than injectable ones, should be given to all patients when feasible.
- Growth factors and oral antibiotics can be used for patients receiving high-risk chemotherapy as primary prophylaxis for febrile neutropenia and empiric oral antibiotics in patients with febrile neutropenia but clinically stable.
- High-dose methotrexate (HDMTX) can be administered in the outpatient setting, replacing IV sodium bicarbonate with oral tablets (adding acetazolamide if needed) ensuring appropriate monitoring. Additionally, oral leucovorin can replace the intravenous form post HDMTX.
- Cyclophosphamide and ifosfamide can be given in the outpatient setting with proper pre-hydration and IV mesna (sodium 2-mercaptoethanesulfonate) dose followed by oral mesna; it needs adequate patient education for monitoring hemorrhagic cystitis signs.
- In non-acute cases, electrolytes should be replaced orally when feasible.

### Recommendation # 5. Delay stem cell transplants if medically feasible

Hematopoietic stem cell transplant patients (HSCT) have an increased risk of contracting COVID-19 due to the increased intensity of chemotherapy regimens and prolonged immunosuppression. The following recommendations are partly adapted from the American Society for Transplantation and Cellular Therapy (ASTCT) and European Society for Blood and Marrow Transplantation (EBMT) recommendations.^63,64^

1. If the patient disease risk allows, postpone all procedures related to HSCT (mobilization, collection, and conditioning):
  - Examples of autologous transplants that can possibly be postponed include multiple myeloma and low-grade lymphomas.
  - Examples of allogeneic transplant that can possibly be postponed include minimal residual disease (MRD) negative ALL, intermediate-risk acute myeloid leukemia (AML) tolerating consolidation, myelodysplastic syndromes (MDS) patients tolerant to transfusion and without excessive blasts, myelofibrosis (MF) patients tolerating transfusions, and benign hematology indications (e.g. sickle cell disease (SCD), thalassemia)
2. For patients with high risk/aggressive disease, and for whom the transplant cannot be postponed:
  - All patients need to be tested for the COVID-19.
  - Patients who do not have respiratory symptoms and no exposure to the COVID-19, can proceed to transplant after self-isolating for 14 days.
  - In a patient who has had close contact with a COVID-19 patient, BMT procedures should be deferred for 14–21 days from the date of last contact. The patient needs to have two negative polymerase chain reaction (PCR) results one week apart before proceeding with any procedures.
  - For patients who are diagnosed with COVID-19, transplant procedures should be deferred until the patient becomes asymptomatic and has two negative PCR results at least one week apart, with a minimum of a 14-day deferral. Also, it is recommended if possible, to use the lowest intensity regimen for such patients.

## Recommendation # 6. Consider intermittent chemotherapy or treatment discontinuation for eligible patients

To mitigate the negative effects of the COVID-19 pandemic, treatment de-escalation should be considered. This approach is common in the oncology practice, especially in the metastatic setting or in patients who are in deep remission. The use of intermittent chemotherapy does not appear to result in inferior outcomes, according to studies conducted on patients with metastatic colorectal cancer.^65,66^ Clinician judgment appears to be a reasonable method when assessing which patients will have good outcomes with de-escalation. More recently, ESMO recommended that in the metastatic breast cancer setting, chemotherapy holidays should be an option following multidisciplinary tumor board discussion and according to patient preference.^11^

Patients with CML, treatment discontinuation of tyrosine kinase inhibitors (TKI) therapy is considered safe with careful monitoring in adult patients who achieve and maintain a long-term major molecular response (MMR). Supporting evidence for this approach, showing that about half of patients who attempt to discontinue their TKI after the maintenance of MMR remain in treatment-free remission (TFR) after five years.^67^

## Recommendation # 7. Activating telemedicine for managing stable cancer patients on oral chemotherapy

The concept of telemedicine was created to improve patients’ access to healthcare providers in rural areas.^68,69^ In oncology, telemedicine can be implemented to remotely manage the patient’s symptoms, medications, and palliative care. Both patients and providers reported high satisfaction rates when virtual clinics were used. However, advanced, yet complex, technologies have to be implemented to deliver optimal care. Thus, multidisciplinary trained teams are an essential step to successfully provide virtual healthcare. Activating virtual oncology clinics for managing stable patients including the ones on oral chemotherapy (e.g. Chronic Myeloid Leukemia (CML), Chronic Lymphocytic Leukemia (CLL), lung cancer, breast cancer, renal cancer, prostate cancer, and hepatocellular carcinoma), during the COVID-19 pandemic crisis is considered as an essential step to reduce the frequency of visits and possible exposure to the virus.

## Recommendation # 8. Applying innovative ideas to minimize patients visits to the pharmacy

### Sending medications by postal carriers to patients’ homes and use of a drive-through medications collection area

To combat potential viral exposure at outpatient pharmacy waiting area, the use of a drive-through medication collection area should be prepared, where patients are notified by phone call when their medications are ready for collection. Some oncology pharmacies may otherwise choose a courier service to deliver prescribed medications to their patients.^70,71^ The pharmacies have to follow drug storage guidelines to ensure that the medicines delivered to patients are packaged and sealed properly. Moreover, pharmacists must ensure that refrigerated medicines are transported in a temperature-controlled container, maintaining the correct temperature as per product specifications during the transit.^72^

### Providing more medications supply to chronic and stable cancer patients while maintaining pharmacy stock

Another strategy to reduce cancer patients’ visits to the outpatient pharmacy is to identify chronic and stable patients on oral chemotherapy. This can be done in collaboration with the patient’s primary physician. After sorting out this category of patients, the pharmacy can dispense more medication supply for longer duration. For example, two to three months of supply instead of one-month quantity. Before adopting this strategy, the pharmacy needs to have proper communication with the supply chain department to avoid any future drug shortages.

### Collaboration between pharmacy departments at oncology centers to continue providing treatments of patients who are affected by the travel restriction

One more initiative can be applied to those patients who are affected by curfew is to establish an agreement among pharmacy departments at different oncology centers to continue providing those categories of patients with their chemotherapy. The SOPA panel suggests that the cancer patients who are affected by travel restriction should be identified by their primary physicians. The appropriate and equipped facility needs to be determined by the pharmacy administration at the oncology centers and the ministry of health (MOH) to ensure proper continuity of care. Moreover, local treating physicians should be identified first then receive detailed medical reports with clear treatment plan from referring physicians. Additionally, a local oncology pharmacist/s should be identified and communicate with the oncology pharmacist/s at the oncology center for any consultations. If chemotherapy is unavailable at the local hospital, the oncology center may ship the drug to local facility if condition permits and safety measures in place.

### Activation of 24 h hotline/on-call service run by oncology pharmacist for consultation and inquires related to cancer therapies

Patient education and counseling through phone can contribute to a positive outcome. It motivates the patients to follow the pharmacotherapeutic regimens and monitoring plans. To mitigate the negative effects of the COVID-19 pandemic, we recommend activating a toll free-24 h hotline so whenever cancer patient is experiencing problems with his or her medications, has a drug information questions, or developed side effects, the patients can call the pharmacist/s who will gather the appropriate data and assess the problems. Then adjust the regimens according to protocols or notify the prescribers for further assessment.

In conclusion, delivering cancer care during the COVID-19 pandemic carries significant challenges. Our paper addresses suggestions to properly manage cancer patients during these difficult times. However, implementing such changes in practice mandates a national collaboration from different sectors to guarantee the quality and continuity of care. The SOPA expert panel developed these recommendations, to help in guiding authorities and caregivers to maintain access to cancer therapy, while minimizing the risk of COVID-19 exposure.
